# Clinical Epidemiological Characteristics and Risk Factors for Severity of SARS-CoV-2 Pneumonia in Pediatric Patients: A Hospital-Based Study in Vietnam

**DOI:** 10.7759/cureus.58371

**Published:** 2024-04-16

**Authors:** Sang N Nguyen, Lam T Vu, Ha T Nguyen, Giang H Dao, Anh Ngoc T Nguyen

**Affiliations:** 1 Pediatrics, Haiphong University of Medicine and Pharmacy, Haiphong, VNM; 2 Otolaryngology, Hanoi Medical University, Hanoi, VNM

**Keywords:** clinical epidemiology, pneumonia, children, covid-19, sars-cov-2

## Abstract

Introduction

Coronavirus disease (COVID-19) is an infectious disease caused by SARS-CoV-2, which can cause organ failure in several organs, cardiac problems, or acute respiratory distress syndrome (ARDS). Identifying clinical epidemiological characteristics and risk factors for complications of COVID-19 allows clinicians to diagnose and treat promptly.

Objectives

This study aims to describe the clinical epidemiological characteristics of COVID-19 and assess risk factors for the severity of SARS-CoV-2 pneumonia in children treated at Haiphong Children’s Hospital.

Methods

A descriptive cross-sectional study was conducted in Haiphong Children’s Hospital, Haiphong, Vietnam, for one year, from January 1, 2022, to December 31, 2022.

Results

In our study, 540 children were evaluated; the male-to-female ratio was 1.48/1; the median age was 23 months (IQR=6-74); Children aged under one year accounted for the highest proportion (n=202; 37.4%); 40 (7.4%) children had underlying illnesses. The number of admitted patients diagnosed with COVID-19 peaked in February 2022. Regarding severity, 380 (70.4%) cases were mild, 136 (25.2%) were moderate, only 24 (4.4%) cases were severe, and no children died. Common symptoms were fever in 483 (89.4%), coughing in 399 (73.9%), and tachypnea in 163 (30.2%) children. Laboratory features: white blood cell count, platelet count, serum CRP, and coagulation test showed little change. Around 116 (21.5%) had lymphopenia and 148 (27.4%) had pneumonia. Patients under one year were approximately 1.64 times more likely to experience pneumonia complications from COVID-19 than those without such a history (OR=1.64, 95%CI = 1.12 - 2.41, p=0.0112). Patients with underlying conditions were approximately 2.08 times more likely to experience pneumonia complications from COVID-19 compared to those without such conditions (OR=2.08, 95%CI =1.08 - 4.02, p=0.0289).

Conclusion

In COVID-19 pediatric patients, the severity of the disease was mild to moderate without any mortality. Children aged under one year accounted for the highest proportion of all COVID-19 patients. This study found that age under one year and underlying illnesses are related to pneumonia in COVID-19 pediatric patients.

## Introduction

In December 2019, a new strain of coronavirus appeared in Wuhan, Hubei, China, and was identified as the cause of an acute respiratory infection epidemic (SARS-CoV-2). The virus then spread rapidly all over China and most countries around the world [1,2]. In February 2020, the International Committee on Taxonomy of Viruses (ICTV) officially named this new strain of coronavirus SARS-CoV-2 (severe acute respiratory syndrome coronavirus 2) [3]. On March 11th, 2020, the World Health Organization (WHO) declared SARS-CoV-2 a global pandemic and named it COVID-19 (coronavirus disease-19) [4]. Variants that display similar mutations as the variants of concern (VOC) but spread less widely are classified as variants of interest (VOI). As of April 1, 2022, there are five VOCs classified by WHO and are designated as Alpha (B.1.1.7), Beta (B.1.351), Gamma (P.1), Delta (B.1.617.2), and Omicron (B.1.1.529) [5].

On January 23, 2020, Vietnam announced the first two cases of SARS-CoV-2 infection [6]. Since then, Vietnam has seen four waves of the pandemic, with the number of cases increasing in the later waves [7-9]. In early 2022, the Omicron variety triggered a rise of SARS-CoV-2 diseases in Vietnam, signifying community transmission [10].

With the rapid worldwide spread of the disease caused by SARS-CoV-2, the number of children infected with COVID-19 has increased dramatically. However, despite having experienced four major outbreaks, there are currently limited reports on clinical epidemiological characteristics and risk factors for the severity of COVID-19 pneumonia in children compared with adults. This study aimed to describe the clinical epidemiological characteristics and the risk factors for the severity of COVID-19 pneumonia in pediatric patients at Haiphong Children’s Hospital from January 1, 2022, to December 31, 2022.

## Materials and methods

Study population

We conducted a retrospective study of 540 children with COVID-19 admitted to Haiphong Children’s Hospital, Vietnam, from January 1, 2022, to December 31, 2022. Criteria for COVID-19 diagnosis were based on the COVID-19 diagnosis of Yüce and colleagues [11]. Haiphong Children's Hospital is one of the largest and oldest pediatric hospitals in North Vietnam.

Inclusion criteria

The study enrolled children under 16 years of age diagnosed with COVID-19 (positive quantitative RT-PCR result for SARS-CoV-2) treated at Haiphong Children’s Hospital.

Exclusion criteria

Children who did not have positive quantitative RT-PCR results for SARS-CoV-2.

Methodology

We reviewed clinical records, laboratory findings, and diagnostic imaging for all 540 children. All patients’ nasopharyngeal swab samples were collected and tested for SARS-CoV-2 using quantitative RT-PCR. All samples were processed simultaneously at the Department of Clinical Laboratory of Haiphong Children’s Hospital.

We classified COVID-19 into four levels based on the severity of symptoms: mild, moderate, severe, and critical. Mild patients only present mild symptoms without radiographic features. Moderate patients present with fever, respiratory symptoms, and radiographic features. Severe patients meet one of three criteria: (a) dyspnea, RR greater than 30 times/min, (b) oxygen saturation less than 93% in ambient air, and (c) PaO2/FiO2 less than 300 mm Hg. Critical patients meet one of three criteria: (a) respiratory failure, (b) septic shock, and (c) multiple organ failure [12].

Fever classification [13]: Low-, medium- and high-grade fever were defined as 37.3-38.0°C, 38.1-39.0°C and >39.0°C, respectively. We used the WHO definition for obesity [14].

Data analysis

The data was analyzed using the Statistical Package for Social Sciences (SPSS) software version 27.0 (IBM Corp., Armonk, NY). Pearson's chi-square test was used to analyze the association between categorical variables. All data are presented as mean ± standard error of the mean.

Ethical approval

Approval for the study was obtained from the Medical Ethics Council of the Haiphong University of Medicine and Pharmacy number 206/QĐ-YDHP, and informed consent was obtained according to the Declaration of Helsinki.

## Results

**Figure 1:**
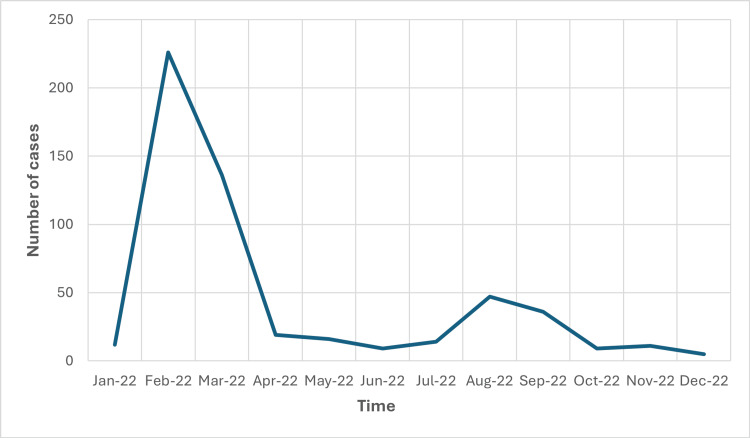
Pandemic curve of COVID-19 cases in a one-year period

There were 540 children admitted due to COVID-19 over one year. The number of admitted patients diagnosed with COVID-19 had one peak occurrence in 02/2022 (Figure 1).

The mean age was 42.6 months, with a median (IQR) of 23 (6-74) months, with a higher number of cases in children under four years old (Table 1). There were 1.48 males for every female. Premature birth and obesity were the two most common underlying diseases seen in COVID-19 children, standing at 16 (3.0%) and 10 (1.9%), respectively. In total, 344 (63.7%) patients lived in rural areas. Additionally, 380 (70.4%) cases had mild COVID-19; no children died in the research period.

**Table 1:** Demographic features of research patients * Our study had no patient with more than one condition.

Characteristics	Number of patients	Percentage
Age group (years)		
<1	202	37.4%
1 – 4	168	31.1%
5 – 9	125	23.1%
10 – 16	45	8.3%
Sex		
Male	322	59.6%
Female	218	40.4%
Location		
Urban	196	36.3%
Rural	344	63.7%
Severity		
Mild	380	70.4%
Moderate	136	25.2%
Severe	24	4.4%
Death	0	0%
Pre-existing comorbidities*	40	7.4%
Premature birth history	16	3.0%
Obesity	10	1.9%
Congenital heart diseases	8	1.5%
β-Thalassemia	3	0.6%
G6PD deficiency	3	0.6%

The clinical characteristics of COVID-19 children are shown in Table 2. Fever was the most prevalent symptom (n=483; 89.4%), followed by cough (n=399; 73.9%). We noted that there were no cases with loss of taste or loss of smell. The investigation results were not specific to COVID-19 patients. Regarding complications, there were 148 cases (27.4%) having pneumonia as a complication of SARS-CoV-2, and 24 cases (4.4%) had severe pneumonia and needed oxygen therapy.

**Table 2:** Clinical features and investigation results of COVID-19 patients

Clinical features and investigation results	Number of patients	Percentage
Fever	483	89.4%
Low grade (37.3 – 38.0 degrees Celcius)	195	40.4%
Medium grade (38.1 – 39.0 degrees Celcius)	153	31.7%
High grade (> 39.0 degrees Celcius)	135	27.9%
Cough	399	73.9%
Tachypnea	163	30.2%
Runny nose	131	24.3%
Tonsil inflammation	103	19.1%
Vomiting	82	15.2%
Diarrhea	59	10.9%
Headache	28	5.2%
Myalgia	21	3.9%
Purpura	16	3.0%
Elevated white blood count	52	9.6%
Decreased white blood count	81	15%
Lymphopenia (< 1.2x10^9^ per liter)	116	21.5%
Elevated serum C-reactive Protein (>12 mg/l)	94	17.4%
Decreased prothrombin time	64	11.8%
Increased prothrombin time	17	3.2%
Decreased activated partial thromboplastin time	25	4.6%
Increased activated partial thromboplastin time	17	3.2%
Decreased fibrinogen	4	0.7%
Increased fibrinogen	10	1.8%
Elevated D-dimer	57	10.5%

Table 3 showed that pre-existing comorbidities and age under one year were risk factors for pneumonia complications in COVID-19 patients. Patients under one year are approximately 1.64 times more likely to experience pneumonia complications from COVID-19 than those without such a history (OR=1.64, 95%CI =1.12 -2.41, p=0.0112). Patients with underlying conditions are approximately 2.08 times more likely to experience pneumonia complications from COVID-19 compared to those without such conditions (OR=2.08, 95%CI =1.08 - 4.02, p=0.0289). No children had cardiac complications.

**Table 3:** Risk factors related to pneumonia related to COVID-19 patients *p-value<0.05 (significant)

Risk factors	Pneumonia related to COVID-19	No Pneumonia	OR (95% CI)	p-value
Premature birth history				
Yes	7 (43.8%)	9 (56.2%)	2.11 (0.77 – 5.78)	0.14
No	141 (26.9%)	383 (73.1%)		
Pre-existing comorbidities				
Yes	17 (42.5%)	23 (57.5%)	2.08 (1.08 – 4.02)	0.0289*
No	131 (26.2%)	369 (73.8%)		
Obesity				
Yes	3 (30%)	7 (70%)	1.14 (0.29 – 4.46)	0.85
No	145 (27.4%)	384 (72.6%)		
Age under one year				
Yes	69 (33.7%)	136 (66.3%)	1.64 (1.12 – 2.41)	0.0112*
No	79 (23.6%)	256 (76.4%)		

## Discussion

Our study described the clinical features and investigation results of children with SARS-CoV-2. It was observed that only children who had symptoms came to the hospital for examination and had positive results with SARS-CoV-2. Therefore, our study had no asymptomatic SARS-CoV-2. For a one-year period, there were 540 children hospitalized in Haiphong Children’s Hospital due to SARS-CoV-2 infection. Most children with COVID-19 had mild clinical symptoms, 70.4% (380/540) and only 24 cases showed severe disease.

We noted that the COVID-19 wave, primarily driven by the Omicron variant, reached its zenith in February 2022. This observation is corroborated by Niha [15] and Ung [10], who also identified February as the peak period for the year 2022.

We observed that the median age of children who were SARS-CoV-2 infected was 23 months, with the majority being under one year of age (37.4%). Our result is similar to Tannis et al.’s study in the USA, with the median age being 22 months [16]. Our median age is much lower compared to other studies. Yılmaz et al. noted that the median age of SARS-CoV-2 patients in Türkiye in 2022 was 92.5 months [17]. COVID-19-associated hospitalization was defined as any person who tested positive for SARS-CoV-2 while admitted to our hospital. So, a large number of asymptomatic patients were not recorded. In Vietnam, parents give their younger children (those under the age of one) more attention. Therefore, whenever their children exhibit any symptoms, such as a fever or cough, they frequently bring them to the hospital for evaluation. In many instances, parents of children older than one only choose at-home treatment rather than sending them to the hospital. That can be attributed to the differences between our study and others. In our study, males were predominant, and the male-to-female ratio was 1.48, which was similar to Dong [2], Tannis [16], and Hardelid [18]. 

In 2022, the two most prevalent symptoms were fever and cough, accounting for 89.4% and 73.9%, respectively, as reported in the majority of research [17,19]. Lu et al. found that the most common clinical symptoms were cough (52%), fever (41%), pharyngitis (46%), diarrhea (9%), and vomiting (6%) [20]. This distinction stems from the fact that most asymptomatic patients were treated at home at the time of our investigation.

In our study, the severity of the disease was primarily mild to moderate, with the proportion of pneumonia being 27.4%, and only 24 cases (4.4%) had severe pneumonia that needed oxygen therapy. Cloete et al. also noted the same findings that most of their patients were mild-to-moderate [21], but the percentage of patients using oxygen therapy was higher than ours (20%).

Regarding laboratory characteristics, we noted that 116 cases (21.5%) had lymphopenia and 70.7% in mild cases. Our result was higher than other studies. Lu et al. found that only 3.5% of their patients had lymphopenia [20]. In Souza's study, 12.9% of patients had reduced lymphocyte counts [4]. We have the same comment with Souza that lymphopenia may not be a reliable predictor of prognosis in children.

According to our research, infants less than a year old or those with underlying medical issues had a higher risk of developing pneumonia. According to Farrar et al. [22], Canadian children under the age of one year had the highest rate of severe COVID-19 (5.4 cases per 100,000 population). Furthermore, Farrar et al. [22] noted that pre-existing comorbidities contributed to the increased risk of severe COVID-19. Prematurity or obesity in children was shown to increase the likelihood of severity in Farrar's study; however, our analysis found no such evidence.

The main limitation of this research is that the data was collected from only one hospital in Haiphong, not an entire country, and may not be representative of another region in Vietnam. Besides, asymptomatic cases were not analyzed, so we cannot estimate the proportion of severity of the disease. Loss of taste or loss of smell could not be assessed in children under one year old.

## Conclusions

Fever and coughing were the most common clinical presentations in admitted pediatric COVID-19 patients. In terms of laboratory characteristics, lymphopenia was present in 21.5% of cases, and other characteristics were not specific to COVID-19 in children. With no death, the disease severity was virtually mild to moderate; just 4.4% were severe. Most COVID-19 patients were younger than one year old. This study discovered a correlation between COVID-19 pneumonia in children under the age of one year and underlying diseases. The findings offer the clinical epidemiological characteristics of COVID-19 in children, which can guide prevention and management strategies.
